# Meiotic dynamics in a unique Australian marsupial provide new insights into the evolution of neo-sex chromosomes in the early stages of differentiation

**DOI:** 10.3389/fcell.2025.1562403

**Published:** 2025-03-20

**Authors:** Laia Marín-Gual, Carolyn J. Hogg, J. King Chang, Andrew J. Pask, Marilyn B. Renfree, Paul D. Waters, Aurora Ruiz-Herrera

**Affiliations:** ^1^ Departament de Biologia Cel·lular, Fisiologia i Immunologia, Universitat Autònoma de Barcelona, Barcelona, Spain; ^2^ Genome Integrity and Instability Group, Institut de Biotecnologia i Biomedicina, Universitat Autònoma de Barcelona, Barcelona, Spain; ^3^ School of Life and Environmental Sciences, The University of Sydney, Sydney, NSW, Australia; ^4^ School of Biotechnology and Biomolecular Sciences, Faculty of Science, The University of New South Wales, Sydney, NSW, Australia; ^5^ School of BioSciences, The University of Melbourne, Parkville, VIC, Australia

**Keywords:** marsupials, meiosis, neo sex-chromosomes, meiotic sex chromosome inactivation, double strand breaks, recombination

## Abstract

Understanding the origin and fate of sex chromosomes has been one of the most intriguing questions in biology. In therian (marsupial and eutherian) mammals, most species are characterized by a heteromorphic XX female XY male sex chromosome system. It is commonly accepted that they originated from a pair of autosomes after gaining a sex-determining function, leading to recombination suppression and subsequent Y chromosome degeneration. Unlike eutherian sex chromosomes which share a pseudo-autosomal region (PAR), the marsupial sex chromosomes are typically tiny and lack any homology. However, there is a lack of empirical evidence on biological systems that represent early stages of sex chromosome differentiation. Here, we describe the meiotic dynamics of an XY_1_Y_2_ system in the greater bilby (*Macrotis lagotis*: family Thylacomyidae) that resulted from a fusion between an autosome and the ancestral X chromosome. We compared the similarities and differences in the regulation of meiosis in two other Australian marsupial species with different sex chromosome systems: the tammar wallaby (*Macropus eugenii*: family Macropodidae) and the fat-tailed dunnart (*Sminthopsis crassicaudata*: family Dasyuridae), both with the ancestral XY system. We performed a cytological analysis of meiotic prophase I, including the study of chromosome synapsis, double strand break formation (as a proxy of recombination) and meiotic sex chromosome inactivation. Our results suggest that the neo-PAR in the greater bilby represents an early stage of differentiation, providing new insights into sex chromosome evolution.

## 1 Introduction

Sex chromosomes are among the most dynamic regions of the genome, fascinating biologists since initial descriptions in hemiptera and mealworms (Wilson, 1905; Muller, 1914; Brush, 1978). Despite the diversity in sex chromosome systems present across the evolutionary Tree of Life (The tree of sex consortium, 2014), it is widely accepted that they originated from a pair of autosomes after gaining a sex-determining function (Muller, 1914; Ohno, 1967). In therian (marsupial and eutherian) mammals, most species are characterized by an XX female XY male sex chromosome system, in which the Y chromosome acquired the dominant testis determining gene *SRY* approximately 180 MYA (Foster et al., 1992). Once this sex-determining factor was established, male beneficial alleles accumulated nearby (in linkage disequilibrium). The subsequent suppression of recombination between the X and Y across this region led to the progressive degeneration of the Y chromosome (Graves, 1995; Graves et al., 1998).

Despite the current degenerated nature of mammalian Y (small in size and gene poor), different hypotheses have been proposed to explain its persistence across species (Toder et al., 2000; Waters and Ruiz-Herrera, 2020; Ruiz-Herrera and Waters, 2022). We recently argued that persistence of Y chromosomes in distantly related mammalian phylogroups can be explained in the context of pseudo-autosomal region (PAR) size, meiotic pairing strategies, and the presence of Y-borne executioner genes that regulate meiotic sex chromosome inactivation (Ruiz-Herrera and Waters, 2022). Under this scenario, persistent Ys are under strong pressure to maintain high recombination rates in the PAR during meiosis to avoid aneuploidies. In the event that executioner protection is lost, the Y chromosome can be maintained by either PAR rejuvenation (extension by addition of autosome material) or gaining achiasmatic meiotic pairing, such as is typically observed in marsupials (Page et al., 2003; Marín-Gual et al., 2022; Valero-Regalón et al., 2023).

**FIGURE 1:**
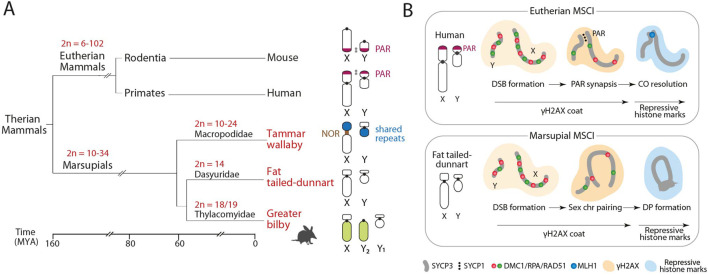
Marsupial sex chromosomes. **(A)** Phylogenetic relationships of the three marsupial species included in the study in relation to eutherians, with the representation of their diploid number (2n) variation and sex chromosome configuration. All marsupials lack the pseudo-autosomal region (PAR). **(B)** Schematic representation of the meiotic sex chromosome inactivation (MSCI) on unsynapsed sex chromosomes in eutherian mammals (upper panel) and marsupials (lower panel). Adapted from Waters and Ruiz-Herrera (2020), Marín-Gual et al. (2022). NOR, Nucleolus Organizer Regions; CO: Meiotic Crossovers; DP, Dense plate.

Sex chromosomes in marsupials are considered to be ancestral to all therian mammals, being generally small in size and lacking the PAR, which was regenerated in the common ancestor of eutherians by the fusion of a large autosomal region (Graves, 1995). Thus, the marsupial X and Y chromosomes do not share a homologous region within which recombination occurs. To prevent aneuploidy stress, marsupials have acquired a faithful achiasmatic mechanism for XY segregation: the dense plate (DP) (Page et al., 2003; Marín-Gual et al., 2022). The DP is a meiotic-specific structure enriched in proteins of the synaptonemal complex formed during the first meiotic division of meiosis (Solari and Bianchi, 1975). This proteinaceous structure allows sex chromosomal segregation during the first meiotic division in the absence of PAR. The formation of the DP occurs in late stages of prophase I, concurrent with meiotic sex chromosome inactivation (MSCI) (Figure 1) (Page et al., 2003; Marín-Gual et al., 2022; Valero-Regalón et al., 2023).

MSCI is a conserved epigenetic silencing program in therian mammals restricted to the heterogametic sex (Richler et al., 1992; Turner et al., 2005). It is a characteristic meiotic feature that includes the accumulation of chromatin modifications along the axes of the X and Y chromosomes as a response to unsynapsed chromatin during prophase I (Turner et al., 2005). This creates a distinct sex chromosome-specific domain (the sex body) at pachytene, which is characterized by the phosphorylation of the histone H2AX (γH2AX), followed by the accumulation of histone marks such as H3K9me3/2, H2A ubiquitylation, and HP1β (Turner et al., 2006; Namekawa et al., 2007) and the absence of active RNA polymerase II, H3K27m1/3, H3K9ac, and H4K16ac (Namekawa et al., 2007; Marín-Gual et al., 2022).

Recent studies in marsupials have shown that different species present divergent strategies for meiotic sex chromosome dynamics (Marín-Gual et al., 2022; Valero-Regalón et al., 2023). This includes species-specific pairing and silencing strategies and different waves of γH2AX accumulation during prophase I, with implications for sex chromosome evolution. However, these initial studies were restricted to marsupial groups with achiasmatic sex chromosomes, such as the tammar wallaby (*Macropus eugenii*, a representative of Macropodidae), the fat-tailed dunnart (*Sminthopsis crassicaudata*, a representative of Dasyuridae), the fat-tailed mouse opossum (*Thylamys elegans*, a representative of Didelphidae) and monito del monte (*Dromiciops gliroides,* a representative of Microbiotheriidae). Whether this pattern is conserved by the addition of new autosomal material to the sex chromosomes remain to be tested.

Here we describe the meiotic dynamics of a neo-sex chromosome in a unique Australian marsupial, the iconic greater bilby (*Macrotis lagotis*). The greater bilby is the only extant member of the family Thylacomyidae, a group of ground-dwelling marsupials known as bandicoots. It is an endangered species with unique biological features that have permitted its adaptation to arid environments, having low metabolic rates and low water turnover (Aslin and Smith, 1980). Importantly, the karyotype of the greater bilby is characterized by the presence of a neo-sex chromosome XY_1_Y_2_ system (Martin and Hayman, 1967), resulting in a diploid number of 2n = 18 in females and 2n = 19 in males.

Neo-sex chromosomes occur by the fusion of autosomes to an existing sex chromosome, creating a new PAR and the potential for new evolutionary strata on the X after suppression of recombination with the new Y. As such, evolutionary recent regions show genomic features typical of incipient sex chromosomes, including low genetic divergence and less advanced dosage compensation (Wright et al., 2016; Ponnikas et al., 2018; Vicoso, 2019). In the greater bilby, the fusion of part of an autosome to the X, but not the non-homologous Y, generated an XY_1_Y_2_ system. Males carry the ancestral Y chromosome (represented by Y_1_), while the unfused autosome homolog is represented as a male-specific Y_2_ chromosome (Figure 1A). Recent genomic data have identified a region of homology (84 Mbp aprox.) between one portion of the bilby neo-X chromosome and the X chromosomes of other marsupial species (Hogg et al., 2024), which is the ancestral X chromosome. This makes it possible to interrogate the meiotic behaviour of a neo-sex chromosome in the context of new genomic resources.

In this study, we report the dynamics of chromosome pairing and MSCI in the greater bilby male germ line. We observed that meiotic pairing dynamics correlate with evolutionary strata in the neo-sex chromosome, with asynaptic regions of neo-XY chromosomes showing meiotic sex chromosome inactivation. Additionally, we detected an accumulation of meiotic DSBs in the ancestral X despite low rates of recombination. Overall, our results suggest that the neo-PAR in the greater bilby represents an early stage of sex chromosome differentiation.

## 2 Materials and methods

### 2.1 Biological samples

Testis tissue from a greater bilby was obtained from an adult male at Taronga Zoo (Sydney, Australia), which was euthanised for medical reasons in 2021. Tammar wallaby males (N = 2, *Macropus eugenii*) were originated from wild populations on Kangaroo Island (South Australia) and held in a breeding colony in Melbourne (Victoria, Australia). Fat-tailed dunnart males (N = 2, *Sminthopsis crassicaudata*) were collected from breeding colonies in Melbourne (Victoria, Australia). All animals were held and tissues collected under appropriate permits, and experiments approved by each Universities Animal Experimentation Ethics Committees in accordance with animal ethics guidelines.

### 2.2 Spermatocyte spreads and immunofluorescence

Spermatocyte spreads were obtained from testicular biopsies as previously described (Garcia-Cruz et al., 2011). Briefly, small pieces of tissue were minced in 1xPBS and incubated for 30 min with 1% lipsol to swell the cells. Then, 4% paraformaldehyde was added and incubated for 2 h in a humid chamber. Slides were finally air-dried, washed with 1% Photo Flo and stored at −20°C until further immunofluorescence experiments.

For immunofluorescence experiments, slides were washed twice with pre-warmed PBST (0.05% Tween 20 in 1xPBS) for 5 min. Primary and secondary antibodies diluted in PBST were added and incubated overnight at 4°C and 1 h at 37°C, respectively. After incubations with antibodies, slides were washed twice with pre-warmed PBST for 5 min and finally mounted with anti-fade solution (Vectashield) containing DAPI (4′,6′-diamidino-2-phenylindole). Antibodies used in the study included: rabbit antibody against SYCP3 (#ab15093, Abcam, dilution 1:100), rabbit antibody against SYCP1 (#ab15090, Abcam, dilution 1:100), mouse antibody against γH2AX (#ab22551, Abcam, dilution 1:200), mouse antibody against RNA polymerase II phosphorylated at serine 5 (#ab5408, Abcam, dilution 1:100), mouse antibody against RPA (#ab111161, Abcam, dilution 1:100), goat antibody against rabbit IgG conjugated to an AlexaFluor® 488 (#ab150085, Abcam, dilution 1:200), goat antibody against mouse IgG conjugated to an AlexaFluor® 555 (#ab150118, Abcam, dilution 1:200) and goat antibody against rabbit IgG conjugated to an AlexaFluor® 555 (#ab150078, Abcam, dilution 1:200).

Axial elements of the synaptonemal complex labelled with anti-SYCP3 were used to classify spermatocytes into the different prophase I stages (leptotene, early zygotene, late zygotene and pachytene) as previously described (Marín-Gual et al., 2022). The proportion of thick (i.e., synapsis) and thin (i.e., asynapsis) SYCP3 filaments were used to distinguish between earlier and later stages of zygotene spermatocytes. SYCP1 staining allowed the identification of synapsed chromosomal regions whereas RPA staining was used as a proxy of DSBs (He et al., 1995; Keeney et al., 1997).

### 2.3 Microscopy and image analysis

IF treated slides were analysed using an epifluorescence microscope equipped with a camera and suitable emission filters. Images were analysed and processed using the public domain software ImageJ (National Institutes of Health, United States; http://rsb.info.nih.gov/ij) and Adobe Photoshop 13.0. RPA foci distribution along chromosomes was analysed using the ImageJ software. For each chromosome, RPA foci on the synaptonemal complex (i.e., SYCP3 signal) were recorded as a relative position (percentage of total length) from the p-arm telomere onwards. Chromosomes were classified according to their length and centromere position. RPA foci distribution from all acrocentric chromosomes (i.e., chromosomes 3, 4, 5, 6 and 8) was merged into one plot since it was not possible to properly differentiate those chromosomes with the SYCP3 signal. Only cells at early pachytene stage were included in the analysis. We analysed at least 15 pachytene spermatocytes.

### 2.4 Statistical analysis

All statistical methods and p-values are indicated in plots or figure legends. Statistical significances for the DSB analysis were determined using two-sided Mann-Whitney U and a p-value <0.05 was considered for statistical significance. All box-and-whisker plots are represented as centre lines (median), box limits (interquartile range; 25th and 75th percentiles) and whiskers (largest and lowest data points inside the first and third quartiles plus 1.5 times the interquartile range).

### 2.5 Chromosome synteny plots

To further validate the X chromosome syntenic regions in the greater bilby, high-level synteny was plotted against other marsupial species using GENESPACE v1.3.1 (Lovell et al., 2022). Marsupial reference genomes used for chromosome synteny plots were the following: *Macrotis lagotis* (Genbank ID: GCA_037893015.1*), Dromiciops gliroides* (Genbank ID: GCF_019393635.1), *Trichosurus vulpecula* (Genbank ID: GCF_011100635.1), *Sarcophilus harrisii* (Genbank ID: GCF_902635505.1) and *Antechinus flavipes* (Genebank ID: GCA_016432865.1).

### 2.6 X-chromosome read depth

Available Illumina data from a male greater bilby was downloaded from NCBI (BioProject PRJNA1049866) and processed as previously described (Hogg et al., 2024). DeepTools bamCoverage was used to calculate read depth in 20 kb windows. A heatscatter of the read depth ratios (versus the PAR mean) was plotted with the R package LSD (Schwalb et al., 2020).

## 3 Results

### 3.1 Meiotic pairing dynamics correlate with evolutionary strata in the neo-sex chromosomes

**FIGURE 2:**
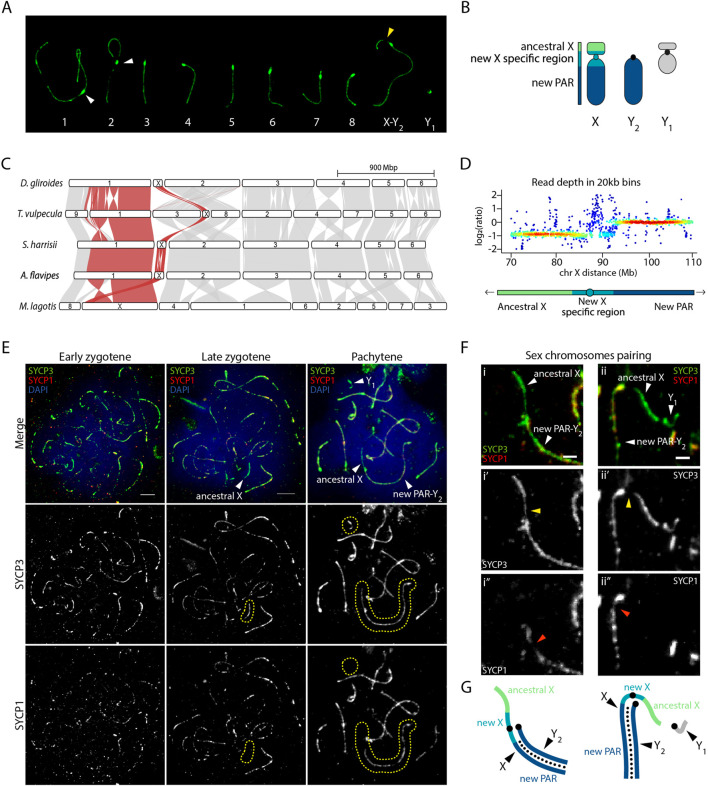
Sex chromosomes pairing in the greater bilby. **(A)** Meiotic karyotype of the greater bilby. The karyotype corresponds to a primary spermatocyte at pachytene labelled with an antibody against SYCP3 (green). White arrows indicate centromeres from chromosomes 1 and 2. The yellow arrow indicates the region on the p-arm of the chromosome X that loads less SYCP3 during meiotic prophase I. **(B)** Schematic representation of sex chromosomes of the greater bilby. **(C)** Genespace synteny plots representing chromosome scaffolds syntenies for different marsupial species. Adapted from Hogg et al. (2024). **(D)** Heatscatter of read depth ratios of male genome sequence data in 20 kb bins of the compound X, demarcated into the ancestral X at half read depth, new X specific region, and a large pseudo-autosomal region (PAR) with full read depth that pairs with the Y_2_ during male meiosis. Adapted from Hogg et al. (2024). **(E)** Primary spermatocytes of the greater bilby labelled with antibodies against SYCP3 (green), SYCP1 (red) and chromatin counterstained with DAPI (blue). Yellow dashed circles demarcate the area where sex chromosomes are located. Scale bar: 10 µm. **(F)** Insets of sex chromosomes pairing in the greater bilby, labelled with antibodies against SYCP3 (green) and SYCP1 (red). Yellow arrows indicate the region on the p-arm of the chromosome X that loads less SYCP3 during meiotic prophase I. Orange arrows indicate the region on the q-arm of the chromosome X where less SYCP1 protein is loaded. Scale bar: 2 µm. **(G)** Schematic representation of sex chromosomes from Figure 2F. Small black dots indicate synapsis (i.e., loading of SYCP1) between the new PAR and Y_2_.

The greater bilby is characterized by a 2n = 18 complement with 9 chromosome pairs (including a large submetacentric X in females), and a 2n = 19 in males, which have a single X and two male-specific Y chromosomes (Martin and Hayman, 1967). Accordingly, 8 autosomic bivalents were detected in male primary spermatocytes: one large metacentric, one large submetacentric and 6 medium size acrocentric chromosomes (Figures 2A, B). The X chromosome corresponds to a large, submetacentric chromosome, whereas the Y_1_ is small, resembling most marsupial species, and then representing the ancestral Y. The Y_2_, on the other hand, is a long telocentric chromosome, with a size and morphology similar to the long arm of the X.

Our analysis detected that the neo-sex chromosome XY_1_Y_2_ system displayed characteristic meiotic features according to evolutionary strata (Figures 2C, D). Read depth analysis showed a new X-specific region with half read depth, whereas most of the new PAR displayed full read depth in males (Hogg et al., 2024) (Figures 2C, D). These two regions exhibited different dynamics during meiosis. At pachynema, when homologous chromosomes are fully synapsed, we detected conspicuous signal of the lateral element of the synaptonemal complex SYCP3 corresponding to XY_1_ chromosomes (Figures 2A, E–G). However, the intensity of the SYCP3 signal was not homogenous along the axes. We detected low SYCP3 intensity in the proximal region of the p-arm, corresponding to the new X specific region (Figures 2A, F). This was validated by the SYCP1 staining (a marker of the transverse element of the synaptonemal complex), which was absent in the whole p-arm of the X chromosome and was slightly reduced in proximal regions of the q-arm, adjacent to the centromere (Figures 2F, G). These results suggest that the X and Y_2_ fully pair along their full length until the regions corresponding to the new X specific region. Moreover, the region corresponding to the ancestral X remain unsynapsed, as revealed by the presence of SYCP3 and absence of SYCP1 (Figures 2F, G). The same applied to the Y_1_, a very small chromosome that appeared to load SYCP3 at pachynema, but not SYCP1 (Figures 2E–G).

Our results show that the region corresponding to the ancestral X and Y chromosomes maintain the meiotic asynaptic features that characterize all marsupials (Page et al., 2005; Marín-Gual et al., 2022; Valero-Regalón et al., 2023). The same applied to the new X-specific region corresponding to peri-centromeric regions.

### 3.2 Asynaptic regions of neo-sex chromosomes show meiotic sex chromosome inactivation

We next investigated whether the presence of asynaptic regions in the greater bilby (ancestral X and Y_1_) was accompanied by an accumulation of γH2AX signal. The phosphorylation of histone H2AX on serine 139 (γH2AX) is a chromatin modification that appears in response to asynapsed chromatin during prophase I, forming the MSUC (meiotic silencing of unsynapsed chromatin). A specialized version of the MSUC is restricted to the sex chromosomes in the form of the meiotic sex chromosome inactivation (MSCI) (Turner et al., 2005). Previous surveys in different marsupial species detected MSCI, distinguished by two waves of γH2AX signalling (Marín-Gual et al., 2022). During the first wave in early and late zygotene, a faint γH2AX signal is present all over the nucleus, which is later restricted to the sex chromosomes during late prophase I, corresponding to the initiation of MSCI (Marín-Gual et al., 2022).

**FIGURE 3:**
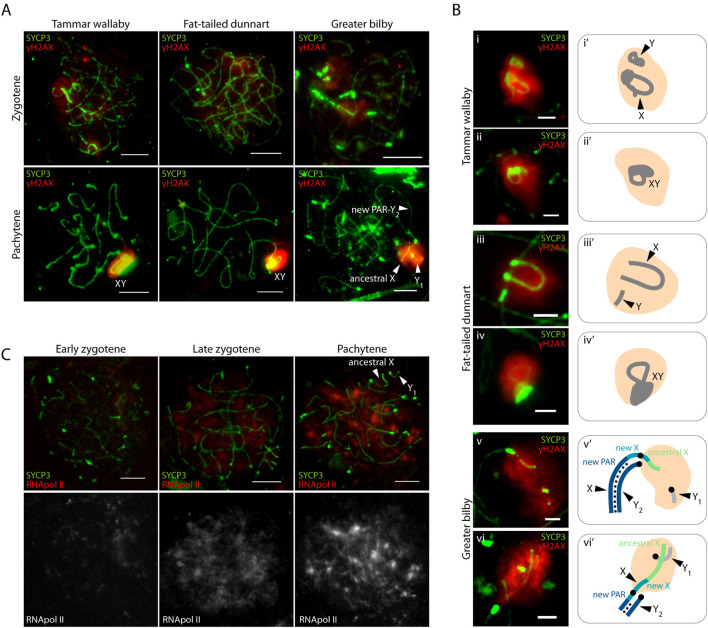
Meiotic sex chromosome inactivation in the greater bilby. **(A)** Primary spermatocytes of the tammar wallaby, fat-tailed dunnart and greater bilby labelled with antibodies against SYCP3 (green) and γH2AX (red). Scale bar: 10 µm. **(B)** Insets of sex chromosomes silencing in the tammar wallaby, fat-tailed dunnart and the greater bilby labelled with antibodies against SYCP3 (green) and γH2AX (red) (i-vi), and their schematic representation (i'-vi’). Scale bar: 2 µm. **(C)** Primary spermatocytes of the greater bilby labelled with antibodies against SYCP3 (green) and RNA polymerase II (red). Scale bar: 10 µm.

Here we detected in the greater bilby the same pattern that is observed in tammar wallaby and fat-tailed dunnart (Figure 3A). γH2AX accumulates on the X and Y early before pairing, at zygotene, and is maintained on unsynapsed sex chromosomes in close proximity during late pachytene (Figure 3A). Whereas the second wave of γH2AX was concurrent with the formation of the DP in both the tammar wallaby and fat-tailed dunnart, we did not detect the presence of the DP in late stages of prophase I in the greater bilby (Figure 3B). Instead, the XY_2_ and Y_1_ approach at pachytene forming a conspicuous γ-H2AX domain without forming a DP (Figure 3B).

Importantly, the γH2AX signal did not span the centromeric region of the X chromosome. Instead, γH2AX staining was restricted to the chromosomal regions corresponding to the ancestral X and the proximal regions of the new X specific region (Figure 3B). This suggests that the centromere can act as barrier for the accumulation of repressive marks that trigger MSCI in neo-XY chromosomes, as was previously suggested for rodents (Deuve et al., 2008).

Moreover, the second wave of γH2AX was accompanied by transcriptional silencing of sex chromosomes in the greater bilby (Figure 3C). As prophase I progressed, transcription increased genome-wide to exception of sex chromosomes. This was reflected by the immunostaining of the phosphorylated RNA pol II (the active form of RNA pol II), which was most predominant in late zygotene, and increased in pachytene (Figure 3C). Therefore, MSCI of asynaptic regions of the new X and the Y_1_ was accompanied by transcriptional silencing.

### 3.3 Accumulation of meiotic DSBs in the ancestral X despite low rates of recombination

**FIGURE 4:**
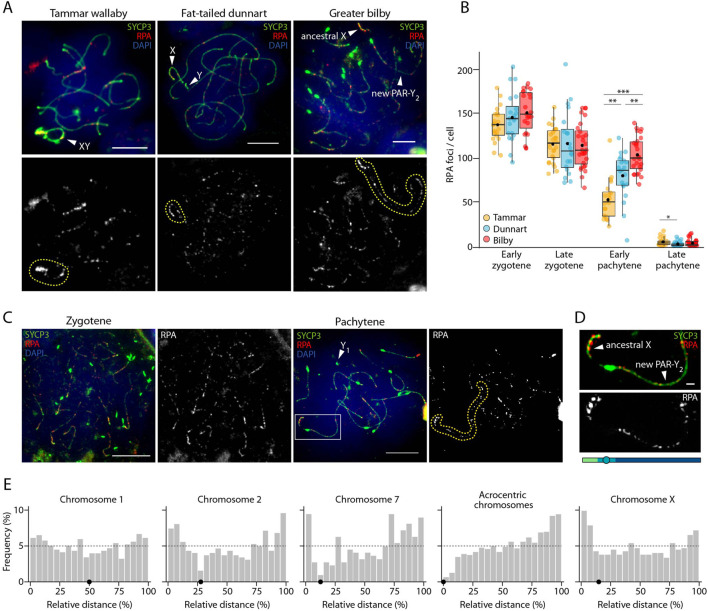
Formation and chromosomal distribution of meiotic DSBs in the greater bilby. **(A)** Primary spermatocytes of the tammar wallaby, fat-tailed dunnart and greater bilby labelled with antibodies against SYCP3 (green), RPA (red) and chromatin counterstained with DAPI (blue). Yellow dashed circles demarcate the area where sex chromosomes are located. Scale bar: 10 µm. **(B)** Boxplot representing the number of RPA foci per cell detected at early zygotene (N = 23 cells), late zygotene (N = 28 cells), early pachytene (N = 26 cells) and late pachytene (N = 30 cells). Data from the tammar wallaby and the fat-tailed dunnart were extracted from previous descriptions (Marín-Gual et al., 2022). Wilcoxon pairwise test (*p < 0.05, **p < 0.01, ***p < 0.001). **(C)** Primary spermatocytes at zygotene and pachytene of the greater bilby labelled with antibodies against SYCP3 (green), RPA (red) and chromatin counterstained with DAPI (blue). Yellow circles demarcate the area where sex chromosomes are located. White square corresponds to the inset of Figure 4D. Scale bar: 10 µm. **(D)** Inset of the X chromosome of the greater bilby labelled with antibodies against SYCP3 (green) and RPA (red). Scale bar: 2 µm. **(E)** Histogram distribution of RPA foci (frequency of RPA foci) along chromosome length of chromosome 1 (N = 23 chromosomes analysed), chromosome 2 (N = 21 chromosomes analysed), chromosome 7 (N = 21 chromosomes analysed), acrocentric chromosomes (N = 98 chromosomes analysed) and chromosome X (N = 21 chromosomes analysed). Black dots correspond to centromeres. Dashed line corresponds to expected frequency for random distribution.

As marsupials are characterized by having low rates of meiotic recombination among mammals (Segura et al., 2013; Marín-Gual et al., 2022), we analysed whether the same applies to the greater bilby. We investigated the dynamics of DSB formation by immunodetection of the recombination proteins RPA (replication protein A) on spermatocyte spreads (Figures 4A, B). RPA can be used as a proxy of meiotic DSBs as it binds to the 3′ strand following DSB formation and consequently accumulates at these sites (He et al., 1995). We compared the pattern of three marsupial species, the tammar wallaby, the fat-tailed dunnart and the greater bilby.

We detected low levels of RPA foci per cell in all three species all through prophase I (Figure 4B). Values were equivalent for all three species in early (151.7 ± 24.1 in the greater bilby, 137.9 ± 19.7 in the tammar wallaby and 146.3 ± 28.2 in the fat-tailed dunnart) (Mann-Whitney test, p > 0.05) and late zygotene (114.4 ± 25.7 in the greater bilby, 116.0 ± 19.7 in the tammar wallaby and 116.5 ± 36.2 in the fat-tailed dunnart) (Mann-Whitney test, p > 0.05). Differences were only observed in early pachytene, where the greater bilby showed higher values (103.7 ± 20.3) than the tammar wallaby (52.5 ± 22.8) (Mann-Whitney test, p < 0.001) and the fat-tailed dunnart (79.9 ± 27.0) (Mann-Whitney test, p < 0.01). Since the greater bilby has a higher number of chromosomes (2n = 19) than the tammar wallaby (2n = 16) and fat-tailed dunnart (2n = 14), differences in RPA foci can be attributable to higher number of late-repaired DSBs. At the end of prophase I (late pachytene), the majority of DSBs are repaired in all three species (2.9 ± 4.1 in the greater bilby, 4.3 ± 4.7 in the tammar wallaby and 1.7 ± 2.5 in the fat-tailed dunnart) (Figure 4B).

Given the high number of RPA foci detected in early pachytene in the greater bilby, we analysed the chromosomal distribution of this marker. The position of each RPA focus was scored for each bivalent in windows of 5% of chromosomal length (Figures 4C–E) (see methods). We detected that RPA foci tend to localize towards terminal chromosomal regions on all chromosomes. In the case of the neo-XY, RPA foci accumulated preferentially in the ancestral X chromosomal region along the SYCP3 axis, displaying larger RPA foci than other regions of the chromosome. Crucially, this chromosomal region corresponds to an asynaptic chromosomal axis (Figure 2G). Since this region does not have homology in males, DSBs are probably repaired using the sister chromatid by non-homologous end-joining mechanisms, as has been described for eutherian mammals (Enguita-Marruedo et al., 2019).

## 4 Discussion

**FIGURE 5:**
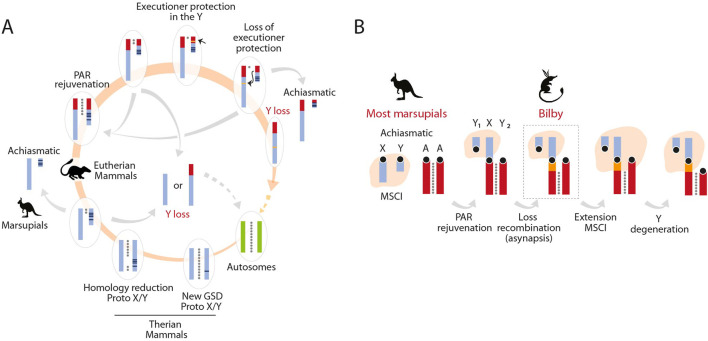
Y chromosome evolution. **(A)** Representation of the cyclical nature of therian Y chromosome evolution. A pair of autosomes can transform into proto-sex chromosomes upon acquiring a sex-determining gene. As beneficial genes accumulate around this gene, the Y chromosome begins to degenerate, reducing homology between the sex chromosomes. At this stage, the pseudo-autosomal region (PAR) can be rejuvenated by the addition of an autosome, as seen in eutherian mammals. This region can then evolve according to the canonical model of sex chromosome evolution. The acquisition of a meiotic executioner gene prevents the Y chromosome from being lost. If the PAR is lost, sex chromosomes can become achiasmatic, as observed in marsupials and some rodents. When the Y chromosome is lost, a new pair of autosomes can become sex chromosomes after acquiring a sex-determining gene, thus completing the cycle. Adapted from Ruiz-Herrera and Waters (2022). **(B)** Proposed Addition-attrition model for the greater bilby. PAR: pseudo-autosomal Region; MSCI: Meiotic Sex Chromosome Inactivation; GSD: Genotypic Sex Determination.

In the cyclical nature of therian Y chromosome evolution, PAR rejuvenation by sex-autosome translocations represents one of the possible trajectories to permit the retention of the Y (Waters and Ruiz-Herrera, 2020; Ruiz-Herrera and Waters, 2022) (Figure 5A). This results in the generation of an extended rejuvenated PAR. According to the addition-attrition model (Graves, 1995; Graves et al., 1998), subsequent suppression of recombination between the X and Y leads to genetic differentiation in new evolutionary strata and the eventual degeneration of the Y chromosome. While this theoretical framework is commonly accepted, the initial steps of sex chromosome differentiation after the addition of a new PAR are still not well understood. This strategy has been observed in some species, from insects (Noronha et al., 2009; Warchałowska-Śliwa et al., 2011; Palacios-Gimenez et al., 2018) to mammals (Vozdova et al., 2016; Gil-Fernández et al., 2020), but empirical evidence is limited. In this context, the neo-sex chromosome XY_1_Y_2_ in the marsupial greater bilby represents a unique model for the study of sex chromosome evolution.

Here we report the meiotic dynamics of a neo-XY chromosomes in the greater bilby, a marsupial species characterized by an XY_1_Y_2_ system. According to the addition-attrition model (Graves, 1995; Graves et al., 1998) and based on the present , the neo-PAR of sex chromosomes of the greater bilby represents an early stage of sex chromosome differentiation, providing new insights into sex chromosome evolution. Specifically, we detected asynapsis between a portion of the Y_2_ and the X (Figure 5B). This is supported by three interconnected observations: (i) asynapsis of the chromosomal region corresponding to the newly added region of the X and Y_2_, (ii) partial presence of MSCI, and (iii) absence of DP.

Our study revealed that the meiotic pairing dynamics in the greater bilby are correlated with evolutionary strata on the neo-sex chromosome. That is, the chromosomal region corresponding to the ancestral X remained unsynapsed at pachynema, as evidenced by the presence of SYCP3 and the absence of SYCP1 in primary spermatocytes. These findings align with previous studies in the African pygmy mouse, which suggested that meiotic synapsis of the neo-PAR can be delayed due to meiotic mechanistic constraints (Gil-Fernández et al., 2020). This delay and asynapsis can eventually lead to a reduction of recombination around centromeric regions that, over evolutionary time, can result in low genetic divergence and less advanced dosage compensation. And this is in fact what we observed in the greater bilby. We detected a reduction of meiotic DSBs at the pericentromeric regions of the X. While the chromosomal region corresponding to the ancestral X strata showed a high number of very bright and large RPA foci (a proxy of meiotic DSBs), the frequency was reduced on the newly added region of the X.

Moreover, we observed the partial presence of MSCI on the X, as measured by the immunodetection of γH2AX deposition. We found that MSCI was present in the X but restricted to the ancestral X region and the proximal portion of the new X specific region. Remarkably, γH2AX deposition did not expanded across the centromere, leaving the small portion of asynaptic region of the new X specific region uncovered. Importantly, these results suggest that the extension of MSCI in response to meiotic asynapsis is a feature that appeared later in evolutionary time (Figure 5B). We expect that given sufficient evolutionary time, lack of recombination in the neo-X-specific region and further extension of MSCI would lead to Y degeneration.

Remarkably, the presence of a DP was not detected in the greater bilby, in contrast with other marsupials. The DP is a meiotic cellular structure that evolved in species such as marsupials (Page et al., 2003; Franco et al., 2007; Marín-Gual et al., 2022; Valero-Regalón et al., 2023) and some eutherian mammals, including gerbils and voles (Ashley and Moses, 1980; Ratomponirina et al., 1986; De La Fuente et al., 2007), to avoid the missegregation of asynaptic sex chromosomes. This structure is enriched in proteins of the synaptonemal complex, such as SYCP3, which is assembled during the first meiotic prophase and is still present at metaphase I (Page et al., 2006). Recent studies have reported divergent strategies during the formation of the DP between marsupial species with different X chromosome morphologies (Marín-Gual et al., 2022), suggesting that the DP is a dynamic structure adapting to different sex chromosome contexts. In the greater bilby, sex chromosomes did not pair but remained associated, with the Y_1_ overlapping the ancestral region of the chromosome X. This observation mirrors previous descriptions in some rodents (Solari and Ashley, 1977; Ashley and Moses, 1980; Gil-Fernández et al., 2021) and voles (Wolf et al., 1988), where achiasmatic sex chromosomes do not form a DP. As for the mechanisms behind this pattern, it has been reported that the histone modification γH2AX persists during the first meiotic division in some achiasmatic sex chromosomes, likely to ensure their association and segregation (De La Fuente et al., 2007; De La Fuente et al., 2012; Gil-Fernández et al., 2020; Gil-Fernández et al., 2021). This model could explain how chromosomes X and Y_1_ from the greater bilby remained associated during late pachytene.

The absence of a DP in the greater bilby suggests that the formation of the DP may have not been necessary after the addition of the new-PAR to an ancestral asynaptic sex chromosome system. In this scenario, the presence of the new-PAR would be sufficient to ensure accurate sex chromosome segregation during the first meiotic division. Given that the DP is mainly formed by proteins of the synaptonemal complex, we anticipate it to be a highly dynamic structure, assembled as an extension of the chromosomal axis in the absence of a PAR. Overall, our results suggest that the neo-PAR in the greater bilby represents an early stage of differentiation, providing new insights into sex chromosome evolution.

## Data Availability

The raw data supporting the conclusions of this article will be made available by the authors, without undue reservation.

## References

[B1] Ashley T. Moses M. J. (1980). End association and segregation of the achiasmatic X and Y chromosomes of the sand rat, Psammomys obesus. Chromosoma 78, 203–210. 10.1007/BF00328392 6993128

[B2] Aslin H. J. Smith M. J. (1980). Marsupials of Australia. Carniv. marsupials bandicoots 2, 292. Melbourne: Lansdowne Editions.

[B3] Brush S. G. (1978). Nettie M. Stevens and the discovery of sex determination by chromosomes. Isis 69, 163–172. 10.1086/352001 389882

[B4] De La Fuente R. Parra M. T. Viera A. Calvente A. Gómez R. Suja J. Á. (2007). Meiotic pairing and segregation of achiasmate sex chromosomes in eutherian mammals: the role of SYCP3 protein. PLoS Genet. 3, e198. 10.1371/journal.pgen.0030198 17983272 PMC2048527

[B5] De La Fuente R. Sánchez A. Marchal J. A. Viera A. Parra M. T. Rufas J. S. (2012). A synaptonemal complex-derived mechanism for meiotic segregation precedes the evolutionary loss of homology between sex chromosomes in arvicolid mammals. Chromosoma 121, 433–446. 10.1007/s00412-012-0374-9 22552439

[B6] Deuve J. L. Bennett N. C. Ruiz-Herrera A. Waters P. D. Britton-Davidian J. Robinson T. J. (2008). Dissection of a Y-autosome translocation in *Cryptomys hottentotus* (Rodentia, Bathyergidae) and implications for the evolution of a meiotic sex chromosome chain. Chromosoma 117, 211–217. 10.1007/s00412-007-0140-6 18094986

[B7] Enguita-Marruedo A. Martín-Ruiz M. García E. Gil-Fernández A. Parra T. P. Viera A. (2019). Transition from a meiotic to a somatic-like DNA damage response during the pachytene stage in mouse meiosis. PLoS Genet. 15, 10074399–e1007535. 10.1371/journal.pgen.1007439 PMC6358097 30668564

[B8] Foster J. W. Brennan F. E. Hampikian G. K. Goodfellow P. N. Sinclar A. H. Lovell-Badge R. (1992). Evolution of sex determination and the Y chromosome: SRY-related sequences in marsupials. Nature 359, 531–533. 10.1038/359531a0 1406969

[B9] Franco M. J. Sciurano R. B. Solari A. J. (2007). Protein immunolocalization supports the presence of identical mechanisms of XY body formation in eutherians and marsupials. Chromosome Res. 15, 815–824. 10.1007/s10577-007-1165-7 17846907

[B10] Garcia-Cruz R. Pacheco S. Brieño M. A. Steinberg E. R. Mudry M. D. Ruiz-Herrera A. (2011). A comparative study of the recombination pattern in three species of Platyrrhini monkeys (primates). Chromosoma 120, 521–530. 10.1007/s00412-011-0329-6 21735165

[B11] Gil-Fernández A. Ribagorda M. Martín-Ruiz M. López-Jiménez P. Laguna T. Gómez R. (2021). Meiotic behavior of achiasmate sex chromosomes in the African Pygmy mouse Mus mattheyi offers new insights into the evolution of sex chromosome pairing and segregation in mammals. Genes (Basel) 12, 1434. 10.3390/genes12091434 34573416 PMC8471055

[B12] Gil-Fernández A. Saunders P. A. Martín-Ruiz M. Ribagorda M. López-Jiménez P. Jeffries D. L. (2020). Meiosis reveals the early steps in the evolution of a neo-XY sex chromosome pair in the African pygmy mouse Mus minutoides. PLoS Genet. 16, e1008959. 10.1371/JOURNAL.PGEN.1008959 33180767 PMC7685469

[B13] Graves J. A. M. (1995). The evolution of mammalian sex chromosomes and the origin of sex determining genes. Philos. Trans. R. Soc. Lond B Biol. Sci. 350, 305–311. 10.1098/rstb.1995.0166 8570696

[B14] Graves J. A. M. Wakefield M. J. Toder R. (1998). The origin and evolution of the pseudoautosomal regions of human sex chromosomes. Hum. Mol. Genet. 7, 1991–1996. 10.1093/hmg/7.13.1991 9817914

[B15] He Z. Henricksen L. A. Wold M. S. Ingles C. J. (1995). RPA involvement in the damage-recognition and incision steps of nucleotide excision repair. Nature 374, 566–569. 10.1038/374566a0 7700386

[B16] Hogg C. J. Edwards R. J. Farquharson K. A. Silver L. W. Brandies P. Peel E. (2024). Extant and extinct bilby genomes combined with Indigenous knowledge improve conservation of a unique Australian marsupial. Nat. Ecol. Evol. 8, 1311–1326. 10.1038/s41559-024-02436-2 38945974 PMC11239497

[B17] Keeney S. Giroux C. N. Kleckner N. (1997). Meiosis-specific DNA double-strand breaks are catalyzed by Spo11, a member of a widely conserved protein family. Cell 88, 375–384. 10.1016/S0092-8674(00)81876-0 9039264

[B18] Lovell J. T. Sreedasyam A. Schranz M. E. Wilson M. Carlson J. W. Harkess A. (2022). GENESPACE tracks regions of interest and gene copy number variation across multiple genomes. Elife 11, e78526. 10.7554/eLife.78526 36083267 PMC9462846

[B19] Marín-Gual L. González-Rodelas L. Pujol G. Vara C. Martín-Ruiz M. Berríos S. (2022). Strategies for meiotic sex chromosome dynamics and telomeric elongation in Marsupials. PLoS Genet. 18, e1010040. 10.1371/journal.pgen.1010040 35130272 PMC8853506

[B20] Martin P. G. Hayman D. L. (1967). Quantitative comparisons between the karyotypes of australian marsupials from three different superfamilies. Chromosoma 20, 290–310. 10.1007/bf00326187 5613986

[B21] Muller H. J. (1914). A gene for the fourth chromosome of Drosophila. J. Exp. Zoology 17, 325–336. 10.1002/jez.1400170303

[B22] Namekawa S. H. VandeBerg J. L. McCarrey J. R. Lee J. T. (2007). Sex chromosome silencing in the marsupial male germ line. Proc. Natl. Acad. Sci. 104, 9730–9735. 10.1073/pnas.0700323104 17535928 PMC1887598

[B23] Noronha R. C. R. Nagamachi C. Y. O’Brien P. C. M. Ferguson-Smith M. A. Pieczarka J. C. (2009). Neo-XY body: an analysis of XY1Y2 meiotic behavior in Carollia (Chiroptera, Phyllostomidae) by chromosome painting. Cytogenet Genome Res. 124, 37–43. 10.1159/000200086 19372667

[B24] Ohno S. (1967). “Sex chromosomes and sex-linked genes,” in Teratology (Berlin: Springer-Verlag), 192. 10.1002/tera.1420040116

[B25] Page J. Berríos S. Parra M. T. Viera A. Suja J. Á. Prieto I. (2005). The program of sex chromosome pairing in meiosis is highly conserved across marsupial species: implications for sex chromosome evolution. Genetics 170, 793–799. 10.1534/genetics.104.039073 15802509 PMC1450418

[B26] Page J. Berríos S. Rufas J. S. Parra M. T. Suja J. Á. Heyting C. (2003). The pairing of X and Y chromosomes during meiotic prophase in the marsupial species Thylamys elegans is maintained by a dense plate developed from their axial elements. J. Cell Sci. 116, 551–560. 10.1242/jcs.00252 12508115

[B27] Page J. Viera A. Parra M. T. De La Fuente R. Suja J. Á. Prieto I. (2006). Involvement of synaptonemal complex proteins in sex chromosome segregation during marsupial male meiosis. PLoS Genet. 2, e136. 10.1371/journal.pgen.0020136 16934004 PMC1557784

[B28] Palacios-Gimenez O. M. Milani D. Lemos B. Castillo E. R. Martí D. A. Ramos E. (2018). Uncovering the evolutionary history of neo-XY sex chromosomes in the grasshopper Ronderosia bergii (Orthoptera, Melanoplinae) through satellite DNA analysis. BMC Evol. Biol. 18, 2–10. 10.1186/s12862-017-1113-x 29329524 PMC5767042

[B29] Ponnikas S. Sigeman H. Abbott J. K. Hansson B. (2018). Why do sex chromosomes stop recombining? Trends Genet. 34, 492–503. 10.1016/j.tig.2018.04.001 29716744

[B30] Ratomponirina C. Viegas-Péquignot E. Dutrillaux B. Petter F. Rumpler Y. (1986). Synaptonemal complexes in Gerbillidae: probable role of intercalated heterochromatin in gonosome-autosome translocations. Cytogenet Cell Genet. 43, 161–167. 10.1159/000132315 3802919

[B31] Richler C. Soreq H. Wahrman J. (1992). X inactivation in mammalian testis is correlated with inactive X-specific transcription. Nat. Genet. 2, 192–195. 10.1038/ng1192-192 1345167

[B32] Ruiz-Herrera A. Waters P. D. (2022). Fragile, unfaithful and persistent Ys—on how meiosis can shape sex chromosome evolution. Hered. (Edinb) 129, 22–30. 10.1038/s41437-022-00532-2 PMC9273583 35459933

[B33] Schwalb B. Tresch A. Torkler P. Duemcke S. Demel C. Ripley B. (2020). Lots of superior depictions [LSD].

[B34] Segura J. Ferretti L. Ramos-Onsins S. Capilla L. Farré M. Reis F. (2013). Evolution of recombination in eutherian mammals: insights into mechanisms that affect recombination rates and crossover interference. Proc. R. Soc. B Biol. Sci. 280, 20131945. 10.1098/rspb.2013.1945 PMC3790489 24068360

[B35] Solari A. J. Ashley T. (1977). Ultrastructure and behavior of the achiasmatic, telosynaptic XY pair of the sand rat (Psammomys obesus). Chromosoma 62, 319–336. 10.1007/BF00327031 891351

[B36] Solari A. J. Bianchi N. O. (1975). The synaptic behaviour of the X and Y chromosomes in the marsupial *Monodelphis dimidiata* . Chromosoma 52, 11–25. 10.1007/BF00285785 1175455

[B37] The tree of sex consortium (2014). Tree of sex: a database of sexual systems. Sci. Data 1, 140015. 10.1038/sdata.2014.15 25977773 PMC4322564

[B38] Toder R. Wakefield M. J. Graves J. A. M. (2000). The minimal mammalian Y chromosome - the marsupial Y as a model system. Cytogenet Cell Genet. 91, 285–292. 10.1159/000056858 11173870

[B39] Turner J. M. A. Mahadevaiah S. K. Ellis P. J. I. Mitchell M. J. Burgoyne P. S. (2006). Pachytene asynapsis drives meiotic sex chromosome inactivation and leads to substantial postmeiotic repression in spermatids. Dev. Cell 10, 521–529. 10.1016/j.devcel.2006.02.009 16580996

[B40] Turner J. M. A. Mahadevaiah S. K. Fernandez-Capetillo O. Nussenzweig A. Xu X. Deng C. X. (2005). Silencing of unsynapsed meiotic chromosomes in the mouse. Nat. Genet. 37, 41–47. 10.1038/ng1484 15580272

[B41] Valero-Regalón F. J. Solé M. López-Jiménez P. Valerio-de Arana M. Martín-Ruiz M. de la Fuente R. (2023). Divergent patterns of meiotic double strand breaks and synapsis initiation dynamics suggest an evolutionary shift in the meiosis program between American and Australian marsupials. Front. Cell Dev. Biol. 11, 1147610. 10.3389/fcell.2023.1147610 37181752 PMC10166821

[B42] Vicoso B. (2019). Molecular and evolutionary dynamics of animal sex-chromosome turnover. Nat. Ecol. Evol. 3, 1632–1641. 10.1038/s41559-019-1050-8 31768022

[B43] Vozdova M. Ruiz-Herrera A. Fernandez J. Cernohorska H. Frohlich J. Sebestova H. (2016). Meiotic behaviour of evolutionary sex-autosome translocations in Bovidae. Chromosome Res. 24, 325–338. 10.1007/s10577-016-9524-x 27136937

[B44] Warchałowska-Śliwa E. Maryańska-Nadachowska A. Grzywacz B. Karamysheva T. Lehmann A. W. Lehmann G. U. C. (2011). Changes in the numbers of chromosomes and sex determination system in bushcrickets of the genus Odontura (Orthoptera: tettigoniidae: Phaneropterinae). Eur. J. Entomol. 108, 183–195. 10.14411/eje.2011.025

[B45] Waters P. D. Ruiz-Herrera A. (2020). Meiotic executioner genes protect the Y from extinction. Trends Genet. 36, 728–738. 10.1016/j.tig.2020.06.008 32773168

[B46] Wilson E. B. (1905). Studies on chromosomes. II. The paired microchromosomes, idiochromosomes and heterotropic chromosomes in hemiptera. J. Exp. Zoology 2, 507–545. 10.1002/jez.1400020405

[B47] Wolf K. W. Baumgart K. Winking H. (1988). Meiotic association and segregation of the achiasmatic giant sex chromosomes in the male field vole (*Microtus agrestis*). Chromosoma 97, 124–133. 10.1007/BF00327369

[B48] Wright A. E. Dean R. Zimmer F. Mank J. E. (2016). How to make a sex chromosome. Nat. Commun. 7, 12087. 10.1038/ncomms12087 27373494 PMC4932193

